# Phosphorus Loading Drives Microalgal Community Changes and Enhances Nutrient Removal in Photobioreactors Treating Synthetic Wastewater

**DOI:** 10.3390/plants15030351

**Published:** 2026-01-23

**Authors:** Ayache Laabassi, Azzedine Fercha, Stefano Bellucci, Alessia Postiglione, Viviana Maresca, Martina Dentato, Asma Boudehane, Laribi Amira, Fatma Z. Saada, Rodeina Boukehil, Zahia Djenien

**Affiliations:** 1 Department of Ecological and Environmental Sciences, Faculty of Natural and Life Sciences, University of Batna 2, Batna 05000, Algeria; a.laabassi@univ-batna2.dz (A.L.); asmaboudehane2@gmail.com (A.B.); laribiamiram2@gmail.com (L.A.); fatmazahrasaada2@gmail.com (F.Z.S.); rodeinaboukehil@gmail.com (R.B.); zahiadjenien2@gmail.com (Z.D.); 2 Laboratory of MCVNAR, Department of Agronomy, Faculty of Nature and Life Sciences, Abbes Laghrour University, Khenchela 40000, Algeria; fercha.azzedine@univ-khenchela.dz; 3 Laboratori Nazionali di Frascati, Istituto Nazionale di Fisica Nucleare, Via E. Fermi 54, 00044 Frascati, Italy; bellucci@lnf.infn.it; 4 Department of Biology, University of Naples Federico II, Complesso Univ. Monte Sant’Angelo, Via Cinthia 4, 80126 Napoli, Italy; alessia.postiglione@inina.it (A.P.); martina.dentato@unina.it (M.D.); 5 Department of Life Sciences, Health and Health Professions, University of Rome “Link Campus”, 00165 Rome, Italy

**Keywords:** phosphorus, microalgae, wastewater treatment, nutrient removal, chlorophyll *a*

## Abstract

Phosphorus is a key nutrient regulating algal growth and eutrophication in aquatic systems, yet its isolated effect on microalgae-based wastewater treatment remains underexplored. This study evaluated how varying phosphorus loads drive microalgal community structure and purification performance in controlled photobioreactors fed synthetic wastewater. The synthetic wastewater was formulated with constant carbon and nitrogen but graded phosphorus at C/N/P ratios of 100/5/1, 100/5/10, and 100/5/20 under 6000 lux, a 14 h photoperiod, and 24 ± 2 °C with a 15-day hydraulic retention time. Monitoring of chlorophyll *a*, pH, total and volatile suspended solids, and algal composition showed that phosphorus enrichment significantly increased chlorophyll *a* (up to 43.9 µg/L at 20 mg P/L) and particulate biomass (TSS and VSS), while pH remained near neutral to slightly alkaline, with no significant differences among the three bioreactors. Although the same core taxa—*Chlorella* spp., *Scenedesmus* spp., *Navicula* spp., and filamentous algae were present across all bioreactors, their relative abundances shifted significantly with phosphorus concentration. A two-way ANOVA confirmed a highly significant interaction between bioreactor (P level) and genus (*p* < 0.001), demonstrating phosphorus-driven changes in the microalgal community. Notably, filamentous cyanobacteria (*Anabaena* spp.) were undetectable in the low- and medium-phosphorus treatments but emerged prominently only at the highest phosphorus level (20 mg/L). Nutrient removal efficiencies peaked in this high-phosphorus bioreactor (C), achieving 85% for bCOD, 78% for nitrogen, and >70% for phosphorus. These results show that phosphorus loading drives predictable shifts in microalgal community composition toward fast-growing algae and cyanobacteria and that these shifts likely contribute to enhanced nutrient removal. The findings support optimization of phosphorus supply and hydraulic residence time in low-cost, sunlight-driven systems to improve polishing performance for small settlements in arid regions.

## 1. Introduction

The rapid increase in domestic, industrial, and agricultural wastewater production is a major driver of aquatic ecosystem degradation worldwide [1,2]. In many low- and middle-income countries, large volumes of effluents are discharged with insufficient treatment, contributing to eutrophication, the spread of pathogens, and the accumulation of emerging contaminants in surface and groundwater used for irrigation and domestic supply. Nutrient-rich discharges, in particular, are strongly associated with the proliferation of algal blooms and long-term degradation of water quality in rivers, lakes, and reservoirs [3,4].

Algeria illustrates this dual challenge of limited water resources and deteriorating water quality [5]. Studies from several northeastern basins report elevated organic loads and nutrient concentrations in rivers and dams receiving urban and industrial effluents, with clear signals of anthropogenic pollution and eutrophication [5,6]. Given the country’s semi-arid climate and high interannual variability in precipitation, there is a pressing need for wastewater treatment technologies that are not only effective in nutrient removal but also compatible with constrained energy and operational budgets [7,8].

Microalgae-based treatment systems have attracted growing interest as a low-energy, resource-oriented option [8]. Recent reviews show that mixed microalgal communities in ponds and photobioreactors can simultaneously remove nitrogen, phosphorus, and organic carbon from municipal and agricultural wastewaters while generating biomass for bioenergy or other bioproducts. When appropriately designed, algal-based processes can achieve tertiary-level polishing of nutrients and pathogens and contribute to circular economy strategies through nutrient recovery and CO_2_ utilization. However, the stability and efficiency of these systems depend critically on nutrient stoichiometry and loading, which control both microalgal metabolism and community composition [8,9].

Phosphorus (P) is a central macronutrient for microalgae, required for ATP, nucleic acids, phospholipids, and intracellular polyphosphate storage. Experimental studies have demonstrated that P availability strongly influences growth rates, biochemical composition, and luxury uptake in both monocultures and mixed algal consortia. At the same time, most engineering-oriented work on algal wastewater treatment has treated phosphorus implicitly, by adopting fixed C/N/P design ratios or by working with real wastewaters whose complex and variable composition makes it difficult to isolate P-specific effects. Even in studies explicitly targeting phosphorus removal and recovery, P is often varied together with other factors (such as light, hydraulic retention time, or nitrogen loading), which limits the ability to attribute changes in community structure and treatment performance to P alone [7,10]

In contrast, the ecological literature provides extensive evidence that nutrient enrichment—and phosphorus loading in particular—drives algal community succession in natural freshwater systems. Long-term observations and experimental work in lakes and reservoirs have documented shifts from diverse communities dominated by diatoms and non-bloom-forming chlorophytes toward assemblages increasingly dominated by fast-growing green algae and, under high nutrient loads and suitable temperatures, by bloom-forming and often toxin-producing cyanobacteria. These successional trajectories are now recognized as hallmarks of eutrophication, yet they have been less systematically explored in engineered, microalgae-dominated reactors, where individual environmental drivers can be tightly controlled [11,12,13].

The present study addresses this gap by using a synthetic wastewater with constant carbon and nitrogen concentrations and systematically varied phosphorus loads. Mixed microalgal communities were cultivated in simple, reproducible photobioreactors operated under controlled light, temperature, and hydraulic retention time, conditions chosen to approximate those of high-rate algal systems while allowing precise manipulation of P input. This design isolates the role of phosphorus as a single regulating factor and enables a direct assessment of how P loading impacts the system as follows:

- Decoupling phosphorus effects through a tightly controlled synthetic medium where only phosphorus loading is varied while carbon and nitrogen remain constant.
- Utilizing straightforward, replicable photobioreactors that simulate natural algal growth conditions (light, temperature, and retention time) while allowing precise manipulation of nutrient inputs.
- Tracking eutrophic community shifts to link phosphorus-driven changes in algal composition (particularly chlorophytes and filamentous cyanobacteria such as *Anabaena* spp.) directly to nutrient removal performance.

Accordingly, the objectives were threefold: (i) to quantify the effect of increasing phosphorus concentration alone on algal biomass and community structure in mixed cultures; (ii) to evaluate the impact of elevated P loads on bCOD, N, and P removal in microalgae-based treatment operated at fixed C and N; and (iii) to determine how phosphorus-driven shifts in algal community composition influence overall purification efficiency, with particular reference to loading conditions typical of municipal and agricultural effluents in arid and semi-arid regions.

## 2. Results

### 2.1. Microalgal Community Composition and Dynamics

**Figure 1:**
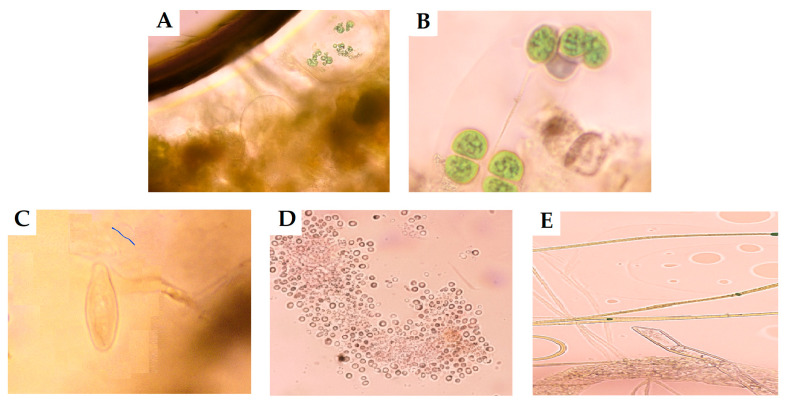
Microscopic images of microalgae isolated from the different bioreactors at an HRT of 15 days. (**A**) Algal mass, (**B**) *Scenedesmus* spp., (**C**) *Navicula* spp., (**D**) *Chlorella* spp., (**E**) filamentous algae.

Microscopic observations and the associated statistical analysis showed a clear phosphorus-driven change in the structure and abundance of microalgal and planktonic communities across the three bioreactors (Table 1 and Table 2, Figure 1). Total algal biomass and cell densities increased significantly from Bioreactors A to C, and community composition shifted in parallel (Table 1). Four major algal taxa were identified: *Chlorella* spp., *Scenedesmus* spp., *Navicula* spp., and filamentous cyanobacteria (*Anabaena* spp.). Phosphorus-driven increases in algal biomass also intensified trophic interactions within the microbial food web, as evidenced by heightened grazing activity, contributing to nutrient recycling.

Although all bioreactors were maintained under similar physical conditions (light, temperature, and mixing), phosphorus concentrations exerted marked effects on algal biomass, species dominance, and overall community structure.

In Bioreactor A (low phosphorus concentration, 1 mg/L), the algal biomass was relatively low but with high species diversity. The genus *Chlorella* spp. was dominant (6.2 ± 1.1 × 10^8^ cells/L), followed by *Scenedesmus* spp. (3.0 ± 0.6 × 10^7^ cells/L) and filamentous forms (2.5 ± 0.5 × 10^7^ cells/L), while *Navicula* spp. was scarce (5.0 ± 1.0 × 10^6^ cells/L). Microscopic observations showed dispersed colonies of *Chlorella* spp. and *Scenedesmus* spp., a pattern consistent with nutrient limitation, although still supporting active primary production. Zooplankton activity was moderate, with *Stylonychia* spp. and other ciliates grazing on suspended microalgae, reflecting an oligotrophic–mesotrophic, well-balanced food web.

In Bioreactor B (medium phosphorus concentration, 10 mg/L), the algal community became denser and more structured, indicating a shift toward eutrophic conditions, with total cell density nearly four times that in Bioreactor A. *Scenedesmus* spp. was dominant (2.2 ± 0.4 × 10^9^ cells/L), followed by *Chlorella* spp. (4.5 ± 0.9 × 10^8^ cells/L) and *Navicula* spp. (3.0 ± 0.6 × 10^8^ cells/L). Filamentous forms (5.0 ± 1.0 × 10^7^ cells/L) indicated the onset of bloom development. Elevated nutrient availability promoted rapid cell division, while *Scenedesmus* spp. formed characteristic coenobia under high light and phosphorus-rich conditions. Zooplankton, mainly ciliates and *Stylonychia* spp., showed strong grazing activity on the increased algal biomass, indicating accelerated but still balanced nutrient recycling.

In Bioreactor C (high phosphorus concentration, 20 mg/L), green microalgae and filamentous forms overwhelmingly dominated, characteristic of a highly eutrophic system. *Chlorella* spp. reached 7.0 ± 1.4 × 10^9^ cells/L—more than ten times its abundance in Bioreactor A. *Scenedesmus* spp. remained abundant (2.0 ± 0.4 × 10^8^ cells/L)—while filamentous cyanobacteria (*Anabaena* spp.) and green filaments increased markedly to 6.0 ± 1.2 × 10^8^ cells/L, becoming major components of mats and aggregates. In contrast, *Navicula* spp. declined sharply (1.2 ± 0.2 × 10^7^ cells/L), likely due to competition from fast-growing chlorophytes and reduced light penetration in the dense biomass. Zooplankton activity peaked, indicating strong trophic pressure and intense predator–prey interactions within the system.

The results of the two-way ANOVA, presented in Table 2, confirmed that both phosphorus level (factor A: F_2,30_ = 3.97, *p* = 0.0296) and taxon identity (factor B: F_3,30_ = 5.14, *p* = 0.0055) had significant effects on cell abundance, with the overall model highly significant (F_5,30_ = 4.67, *p* = 0.0028). Together, these results demonstrate a statistically supported, phosphorus-driven change from a lower-biomass, more even assemblage at low P to a high-biomass, chlorophyte- and filament-dominated community under eutrophic conditions.

Overall, the progression from Bioreactors A to C demonstrates a clear phosphorus-driven shift in community structure within the microalgal community, evolving from a relatively diverse but low-biomass assemblage at low phosphorus levels to a high-biomass, bloom-like community under elevated nutrient conditions. Although species richness remained relatively stable, increasing phosphorus availability intensified the dominance of fast-growing green algae, particularly *Chlorella* spp. and *Scenedesmus* spp., and ultimately promoted the proliferation of filamentous forms and cyanobacteria (e.g., *Anabaena* spp.) at the highest concentrations—patterns typical of eutrophic systems. This change in algal density and dominance highlights the central regulatory role of phosphorus in shaping microalgal composition, productivity, and trophic interactions within the microbial food web, consistent with classical nutrient-enrichment models, and indicates a parallel rise in metabolic activity and nutrient-processing capacity, directly linked to enhanced purification performance examined in the following sections.

### 2.2. Physicochemical Results

**Figure 2:**
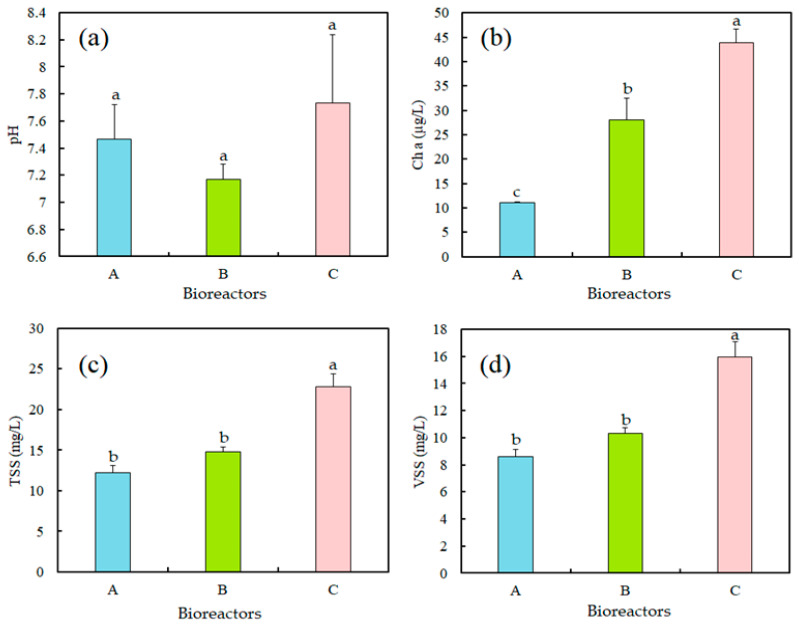
Physicochemical results at the outlet of the three bioreactors. Mean values and standard deviation are represented. (**a**) pH values, (**b**) chlorophyll *a* concentration (µg/L), (**c**) total suspended solids (TSS, mg/L), and (**d**) volatile suspended solids (VSS, mg/L). Data are presented as mean ± standard deviation (*n* = 3). Different lowercase letters above bars indicate significant differences among bioreactors (*p* < 0.05, Tukey’s HSD test).

After ecosystem stabilization, bioreactor performance was monitored through physicochemical analyses. The results obtained are summarized in Table 3 and Figure 2.

#### 2.2.1. Hydrogen Potential (pH)

In this experiment, the initial pH values of the synthetic culture medium were around 7. After 15 days, pH values increased slightly, reaching 7.47 and 7.17 for Bioreactors A and B, respectively, and 7.73 for Bioreactor C (Table 3, Figure 2a). Statistical analysis showed no significant difference in pH among the three bioreactors (F = 2.19, *p* = 0.193, CV = 4.45%), indicating stable conditions throughout the experiment.

#### 2.2.2. Chlorophyll *a*

The highest chlorophyll *a* concentration was recorded in Bioreactor C (43.9 µg/L), which received 20 mg/L of phosphorus, reflecting strong algal proliferation (Table 3, Figure 2b), notably of *Chlorella* spp. and *Anabaena* spp. Bioreactor B exhibited an intermediate value (28.02 µg/L), whereas Bioreactor A, supplied with the lowest phosphorus supply (1 mg/L), showed the lowest chlorophyll *a* concentration (11 µg/L). These differences were highly significant (F = 86, *p* < 0.0001, CV = 11.12%), confirming the strong influence of phosphorus availability on algal growth.

#### 2.2.3. Total Suspended Solids and Volatile Suspended Solids

Total suspended solids (TSS; Figure 2c), consisting primarily of microalgal biomass, were lowest in Bioreactor A (12.26 mg/L) and highest in Bioreactor C (22.8 mg/L). Volatile suspended solids (VSS) followed a similar trend, ranging from 8.57 mg/L in Bioreactor A to 15.96 mg/L in Bioreactor C. Statistical analysis indicated significant differences among bioreactors for both TSS (F = 75.63, *p* < 0.0001, CV = 6.61%) and VSS (F = 75.16, *p* < 0.0001, CV = 6.64%). The VSS/TSS ratio showed slight but significant variation (F = 5.07, *p* = 0.038, CV = 0.03), suggesting comparable proportions of organic material across the bioreactors (Figure 2d).

### 2.3. Nutrient Removal Efficiency

To assess the effect of phosphorus-driven shifts in microalgal community composition on wastewater treatment performance, purification efficiency was evaluated by calculating the removal efficiencies of the main wastewater parameters. These efficiencies were determined from the difference between the initial concentrations in the influent and the final concentrations in each bioreactor after 15 days of hydraulic retention. The corresponding data are presented in Table 4.

Bioreactor C, which received the highest phosphorus load (20 mg/L), exhibited the greatest removal efficiencies for bCOD (~85%), nitrogen (~78%), and phosphorus (~71%). All these values were significantly higher (*p* < 0.0001) than those in Bioreactors B and A. The improved nutrient removal corresponded with the highest algal biomass (Chl. *a*, TSS, VSS) and a distinctive phosphorus-enriched community composition. This community was dominated by fast-growing chlorophytes (*Chlorella* spp. and *Scenedesmus* spp.) and included the filamentous cyanobacterium *Anabaena* spp. The elevated biomass enhanced nutrient assimilation capacity, while the occurrence of *Anabaena* spp. suggested a potential contribution to biological nitrogen fixation. Additionally, the dense, highly productive community increased oxygen production, thereby supporting more efficient heterotrophic degradation of organic matter (bCOD).

### 2.4. Inter-Parameter Relationships

**Figure 3:**
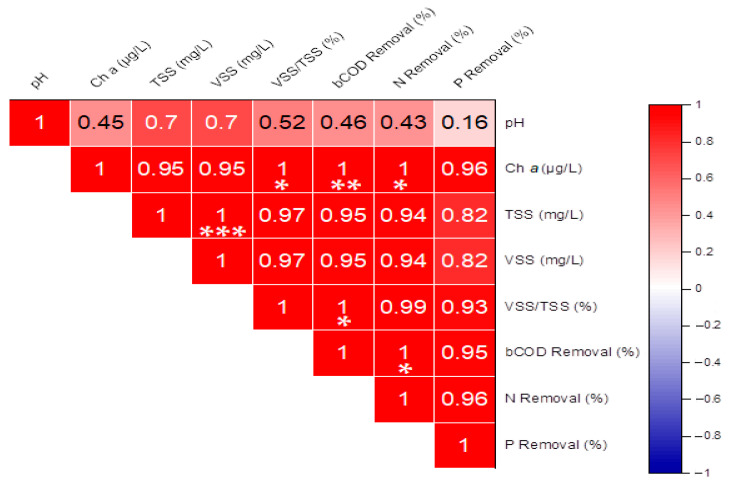
Pearson correlation among the physicochemical water quality parameters. *, significant at *p* ≤ 0.05; **, significant at *p* ≤ 0.01; ***, significant at *p* ≤ 0.001.

Correlation analysis (Figure 3) revealed strong positive associations among key physicochemical indicators of algal activity. Chlorophyll *a* showed very high correlations with TSS and VSS (r = 0.95 for each, *p* < 0.001), indicating that most suspended solids were of biological origin. In contrast, pH exhibited only a moderate correlation with chlorophyll *a* (r = 0.45, *p* > 0.05), while its relationships with TSS and VSS were of similar magnitude but statistically non-significant. The removal efficiencies of bCOD, nitrogen, and phosphorus were all strongly and significantly intercorrelated (r ≥ 0.93, *p* < 0.01); however, these performance indicators showed only weak associations with pH. Taken together, these findings highlight the central role of phytoplankton biomass in determining the system’s particulate load, whereas water alkalinity (pH) and nutrient removal performance exhibited weaker and largely non-significant direct associations with chlorophyll *a* and suspended solids.

## 3. Discussion

This study investigated phosphorus loading effects on microalgal community structure and wastewater treatment performance using controlled photobioreactors. Results revealed a clear phosphorus-driven change from diverse, low-biomass assemblages (Bioreactor A: 1 mg P/L) to *Chlorella*-dominated systems with substantial filamentous cyanobacteria (*Anabaena* spp.) at elevated phosphorus concentrations (Bioreactor C: 20 mg P/L). Critically, this structural shift directly determined treatment efficiency: bCOD (84.9%), nitrogen (77.8%), and phosphorus (71.2%) removal in Bioreactor C significantly exceeded Bioreactor A (68.5%, 62.1%, and 45.3%; *p* < 0.0001). This discussion interprets mechanistic linkages between phosphorus-driven community composition and functional capacity for nutrient removal through species-specific physiological traits.

The shifts in the relative abundance of the dominant genera, particularly the emergence of the filamentous cyanobacterium *Anabaena* as a major component only at the highest P level, clearly indicate a phosphorus-driven restructuring of microalgal community structure. This trend—towards increased density and dominance by fast-growing chlorophytes (*Chlorella*, *Scenedesmus*) and, under high nutrient loads, cyanobacteria—aligns with established paradigms of eutrophication, in which nutrient enrichment can reduce diversity and favor competitive taxa [11,12]. The nearly monospecific communities observed here are typical of polluted or nutrient-enriched waters, where a few competitive species can dominate, thereby reducing overall diversity [14,15]. The dominance of *Chlorella* spp. and *Scenedesmus* spp. across all bioreactors, along with their prolific growth at higher P loads, aligns with their known r-selected life strategy, characterized by rapid nutrient acquisition and accelerated biomass accumulation under resource-replete conditions [16,17]. The exclusive emergence of *Anabaena* spp. in Bioreactor C (20 mg P/L) is particularly significant. Cyanobacteria like *Anabaena* spp. often outcompete green algae under high-P and high-temperature conditions (24 ± 2 °C in our study) due to their more efficient phosphorus storage mechanisms, including the formation of polyphosphate bodies [18,19,20,21]. Recent studies further indicate that high phosphorus availability boosts cyanobacterial competitiveness through enhanced activity of photosynthetic enzymes (e.g., Rubisco, ATP synthase) and superior phosphorus uptake efficiency [22,23], whereas green algae experience photosynthetic downregulation once luxury P uptake becomes saturated [24]. This P-driven change directly impacted the system’s particulate load, as evidenced by the strong positive correlation (r = 0.95, *p* < 0.001) between chlorophyll *a* and suspended solids (TSS/VSS), confirming that the majority of the particulate matter was of biological origin [25,26].

The significant increase in chlorophyll *a* and biomass (TSS and VSS) with increasing phosphorus load underscores the fundamental role of P in photosynthesis and cellular metabolism. Phosphorus is an integral component of ATP, nucleic acids, and phospholipids [27,28]. Its abundance alleviates metabolic constraints, enabling enhanced synthesis of chlorophyll *a* and supporting higher rates of cell division [29,30]. Our results, showing a near fourfold increase in chlorophyll *a* from the lowest to the highest P level, are consistent with previous studies that report a stimulating effect of phosphorus on algal pigment production and biomass yield [31,32]. Conversely, phosphorus deficiency is known to directly impact the key photosynthetic enzyme Rubisco, limit CO_2_ fixation [9], and trigger lipid accumulation as a stress response [33]. The stable, slightly alkaline pH across all bioreactors was within the optimal range for species like *Chlorella vulgaris* [34,35,36] and for diatoms such as *Navicula* spp. [37], preventing the negative impacts of acidification on biodiversity [38,39] and likely facilitating the availability of inorganic carbon for photosynthesis [40,41].

The experimental conditions, particularly the continuous high light intensity (~6000 lux) and absence of agitation, further shaped the system’s dynamics. The high light intensity promoted high photosynthetic rates and influenced chlorophyll *a* concentration, as *Chlorella* spp. species are known to acclimate to light intensity [42,43,44,45]. The light requirement is a crucial factor limiting the productivity of photosynthetic cultures [46], and the range used depends on the species [47]. The absence of water agitation likely contributed to the observed sedimentation and the formation of dense algal masses—a phenomenon noted in stagnant, nutrient-rich waters, where phytoplankton can settle out of the water column [48,49,50,51]. Phosphorus-driven increases in algal biomass also triggered trophic cascades within the microbial food web, intensifying grazing activity and reflecting a multi-trophic level ecosystem [52,53]. The temperature regime of 24 ± 2 °C was optimal for the proliferation of a diverse range of algae, including the cyanobacterium *Anabaena* spp., which typically thrives between 15 and 30 °C [54]. Temperature is a key factor controlling metabolic rates, CO_2_ fixation [55], and phytoplankton growth, which is generally slower in colder waters [56]. It affects the cell cycle, with lower temperatures prolonging division [57] and higher temperatures potentially enhancing bacterial activity and leading to oxygen depletion [58,59,60]. Overall, the controlled environmental conditions maintained a balance conducive to robust photoautotrophic growth [61,62,63,64].

The enhanced nutrient removal efficiency in Bioreactor C (~85% bCOD, ~78% N, ~71% P) reflects a synergistic interaction of multiple biochemical processes stimulated by high phosphorus availability.

The phosphorus removal efficiency increased from 45.3% to 71.2% with higher P loading, reflecting the combined effects of biological uptake and physicochemical processes. Microalgae efficiently assimilate inorganic phosphorus (PO_4_^3−^) for metabolic requirements and store it as polyphosphate, a mechanism well documented in chlorophytes and cyanobacteria such as *Chlorella* spp., *Scenedesmus* spp., and *Anabaena* spp. [65,66,67,68,69]. These organisms can also utilize organic phosphorus through enzymatic hydrolysis [66]. The dominance of fast-growing chlorophytes and filamentous cyanobacteria in Bioreactor C indicates a phosphorus-driven change toward a high-biomass community with enhanced nutrient-processing capacity. Moreover, intensified photosynthetic activity led to an average pH of 7.73, which can shift carbonate equilibria and promote the co-precipitation of phosphorus with divalent cations (e.g., Ca^2+^ and Mg^2+^) as phosphate-bearing minerals [51,52,53,54,55,56,57,58,59,60,61,62,63,64,65,66,67]. Consequently, the superior phosphorus removal observed in Bioreactor C (~71%) likely resulted from maximized biological assimilation into a larger algal biomass combined with favorable conditions for physicochemical precipitation, consistent with previous reports linking sediment phosphorus dynamics to algal activity [68,69].

The removal of biodegradable COD is generally associated with aerobic processes mediated by heterotrophic microorganisms, and the elevated bCOD removal (84.9%) observed in the high-P bioreactor highlights a critical synergy within the photobioreactor system. Under phosphorus-replete conditions, the development of dense microalgal biomass promotes oxygen release during photosynthesis [58]. While this may support aerobic processes, organic matter degradation can proceed under both aerobic and anaerobic conditions, and the specific contribution of heterotrophic bacteria in this study was not quantified. Phosphorus availability is a key factor regulating microbial metabolic activity, which helps explain the enhanced mineralization capacity observed under high-P conditions [70,71]. This oxygen-mediated interaction results in a highly efficient carbon-removal consortium, a principle that underpins many algal-based wastewater treatment systems [72,73].

Nitrogen removal occurred via multiple complementary pathways and was highest in the community dominated by *Chlorella* spp. and *Scenedesmus* spp., notably supplemented by the diazotrophic cyanobacterium *Anabaena* spp. The primary removal mechanism was direct assimilation into algal and bacterial biomass, as evidenced by the increased TSS and VSS values [74,75]. In addition, the presence of *Anabaena* spp. introduced a potentially significant secondary pathway through biological nitrogen fixation. Although nitrate was sufficiently available under the experimental conditions, the establishment of a diazotroph suggests a competitive advantage under high-phosphorus conditions and indicates that, in nitrogen-limited scenarios, this community could partially self-supply nitrogen, thereby further enhancing treatment performance [76]. The convergence of assimilation by the entire microbial community and the potential for nitrogen fixation accounts for the highest nitrogen removal efficiency observed in the most complex and productive bioreactor community. It should be noted that while heterotrophic bacteria undoubtedly play a role in nutrient cycling within the bioreactors, their abundance and specific activities were not measured in this study.

Our findings have direct implications for the design and operation of microalgae-based treatment systems. The clear dose–response relationship demonstrates that maintaining phosphorus concentrations above growth-limiting levels is a powerful lever to enhance treatment performance [72]. By shaping a productive community of fast-growing green algae and functionally diverse cyanobacteria, phosphorus loading directly boosts the system’s purification capacity. The high VSS/TSS ratio (≥70%) across all bioreactors confirms that the systems were dominated by organic, biologically active matter, which is crucial for effective biological treatment [77].

However, this strategy requires careful management. The very high biomass productivity necessitates efficient harvesting protocols to prevent the re-release of nutrients upon cell decay and to realize the potential for resource recovery (e.g., biofertilizers) [78,79,80]. The shift towards cyanobacteria, while beneficial for nutrient removal in this controlled study, must be monitored in full-scale systems due to the potential for toxin production and the role of some genera in forming harmful algal blooms [81]. Therefore, optimization of phosphorus supply involves balancing treatment efficiency with operational stability and environmental safety. Future work should explore the precise dosing thresholds that maximize performance without leading to undesirable cyanobacterial blooms and investigate the integration of these systems with biomass valorization pathways [82]. Ultimately, controlling phosphorus availability is a key strategy for shaping productive microalgal communities that enhance the treatment performance of nature-based wastewater systems [83,84,85,86]. It is important to note that while higher phosphorus loading improved removal efficiency, the absolute residual phosphorus concentration in the effluent of Bioreactor C remained higher than in lower-P treatments. Thus, optimization of P input must balance removal efficiency with final effluent quality to avoid downstream eutrophication.

## 4. Materials and Methods

### 4.1. Experimental Protocol

The reference water for treatability studies is typically urban wastewater, as it is the most extensively documented. However, this reference is imprecise in practice because wastewater composition is highly variable, affected by time of collection, day, and weather events such as rainfall [70]. To achieve a meaningful nutrient balance, a standard C/N/P ratio of 100/5/1 is commonly recommended.

For this study, basic synthetic wastewater was selected to ensure experimental control. Given the potential of nutrients to limit phytoplankton growth, each experimental nutrient solution was supplemented with glucose (C_6_H_12_O_6_) as a carbon source, sodium nitrate (NaNO_3_) for nitrogen, and sodium hydrogen phosphate (Na_2_HPO_4_) for phosphorus.

The experimental protocol began with a five-day stabilization period during which the bioreactors, initially filled with water, were carefully monitored to minimize contamination risks. After stabilization, system performance was assessed through continuous monitoring of chlorophyll *a* concentration, pH, total suspended solids (TSS), and volatile suspended solids (VSS), as well as the identification and enumeration of algal populations.

**Figure 4:**
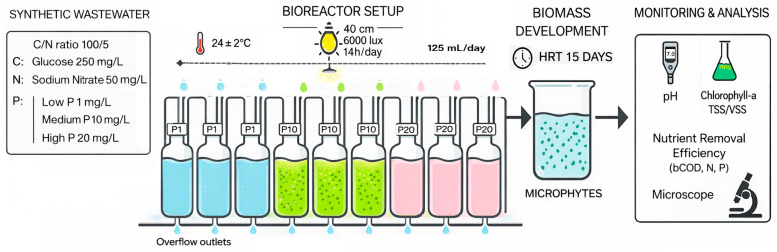
Experimental design.

The reactor system consisted of three parallel sets of polyethylene bioreactors (nine reactors in total), with each set corresponding to one phosphorus level (A, B, and C). Each bioreactor held 1 L and was equipped with an overflow outlet to maintain constant volume. They received a daily supply of 0.125 L nutrient solution via drip feeding, resulting in a theoretical hydraulic retention time of 15 days (Figure 4). Three independent replicates were maintained for each phosphorus treatment. Illumination was provided by halogen lamps placed 40 cm above the water surface, yielding an intensity of 6000 lux; the setup was wrapped in aluminum foil to enhance light distribution. A programmable timer maintained a photoperiod of 14 h. All bioreactors were supplied with the same nutrient solution, differing only in phosphorus concentration according to the tested conditions. The input solutions are as follows:

- Bioreactor A (C/N/P: 100/5/1):
  ○ 250 mg/L of glucose or 100 mg/L of C;
  ○ 30 mg/L of sodium nitrate or 5 mg/L of N;
  ○ 5.5 mg/L of sodium hydrogen phosphate or 1 mg/L of P.
- Bioreactor B (C/N/P: 100/5/10):
  ○ 250 mg/L of glucose or 100 mg/L of C;
  ○ 30 mg/L of sodium nitrate or 5 mg/L of N;
  ○ 61.33 mg/L of sodium hydrogen phosphate or 10 mg/L of P.
- Bioreactor C (C/N/P: 100/5/20):
  ○ 250 mg/L of glucose or 100 mg/L of C;
  ○ 30 mg/L of sodium nitrate or 5 mg/L of N;
  ○ 122.6 mg/L of sodium hydrogen phosphate or 20 mg/L of P.

The photobioreactors were inoculated with microalgae-rich water collected from a eutrophic pond on the campus of the University of Batna 2 in late spring 2025 (May, ambient, 22–28 °C), when green algae dominated. Prior to inoculation, the native microalgal community was characterized via light microscopy using contemporary taxonomic keys that are available at the AlgaeBase (https://www.algaebase.org, accessed on 12 May 2025) [87] and was found to be dominated by the green algae *Chlorella* spp. and *Scenedesmus* spp. with an initial biomass concentration of approximately 0.5 g/L (dry weight). All three bioreactors were inoculated simultaneously with this water at a standardized volume ratio of 10% (*v*/*v*), ensuring identical initial community composition and biomass across all experimental units. The experiment was conducted with three replicate bioreactors for each phosphorus level condition. The various feeding solutions were prepared in advance and stored in a refrigerator to prevent any alterations in their composition. Throughout the experiment, the average temperature corresponded to that of the laboratory, maintained at 24 ± 2 °C.

At the end of the experiment, samples were collected in sterile 1 L screw-cap bottles. Each sample was labeled, stored in a dark cooler, and promptly transported to the laboratory. Upon arrival, samples were kept at 4 °C until analysis to preserve their integrity. Physicochemical analyses were conducted to determine pH, total suspended solids (TSS), and volatile suspended solids (VSS). Additionally, algal species present in the samples were identified and enumerated. The samples were then filtered to isolate the algal biomass for the quantification of chlorophyll *a*.

### 4.2. Study Technique

#### 4.2.1. pH Measurement

The pH was measured by directly immersing the combined electrode into each sample. A precision pH meter (model HI 98230, Hanna Instruments, Woonsocket, RI, USA) was used for this analysis.

#### 4.2.2. Total Suspended Solids (TSS)

The water sample was first filtered, and the residue was then dried in an oven at 105 °C for 24 h. The weight of the dried residue was determined by differential weighing using an electronic balance.

#### 4.2.3. Volatile Suspended Solids (VSS)

The residue remaining after incineration of the sample in a furnace at 525 °C for 2 h represented the mineral content. Volatile suspended solids (VSS) were then estimated as the difference in weight between the total suspended solids (TSS) and the mineral content.

#### 4.2.4. Chlorophyll *a* Assay

First, 250 mL of raw water was filtered through a Whatman GF/C filter (Whatman International Ltd., Maidstone, UK).

The filter was then recovered, and pigments were extracted by dissolving it in 15 mL of 90% acetone. The solution is stored at 4 °C in the dark overnight to ensure complete chlorophyll *a* extraction. Subsequently, the suspension was centrifuged at 3000 rpm for 20 min to obtain a particle-free extract. Absorbance of the extract was measured at 630 nm, 645 nm, and 663 nm using a UV–visible spectrophotometer (model UV-2450, Shimadzu Corporation, Kyoto, Japan). Chlorophyll *a* concentrations were calculated following the SCOR-UNESCO equation:

$$\mathrm{Chlorophyll}\;a\;(\mu g/L)=\frac{\left[\left(11.64\times {OD}_{663}\right)-\left(2.16\times {OD}_{645}\right)-\left(0.1\times {OD}_{630}\right)\times v\right]}{V\times l}$$

where **OD**—optical density, v—volume of the acetone extract in mL, **L**—optical path length in cm, and **V**—volume of the filtered sample in liters.

### 4.3. Study of Algal Microflora

Algal growth was assessed by measuring total suspended solids (TSS) and chlorophyll *a* content. Phytoplankton populations were observed using a light microscope (BX41, Olympus, Tokyo, Japan) with gridded slides and coverslips (Superior; Paul Marienfeld GmbH Co. KG, Lauda-Königshofen, Germany). Microalgal cell counting was performed using a Malassez counting chamber (Superior; Paul Marienfeld GmbH Co. KG, Lauda-Königshofen, Germany). Multiple observations were conducted for each tank to ensure ecosystem stability before proceeding with algal species identification and rough estimation of cell abundance.

### 4.4. Calculation of Nutrient Removal Efficiencies

The removal efficiencies for biodegradable chemical oxygen demand (bCOD), nitrogen (N), and phosphorus (P) were calculated based on the difference between the initial concentration in the feed solution (*C* initial) and the final concentration in the bioreactor effluent after the 15-day hydraulic retention time (*C* final). The removal percentage (*R*) for each parameter was determined using the following formula:

$$R\left(\%\right)=\frac{\left(Cinitial-Cfinal\right)}{Cinitial}\times 100$$

where ***C*initial**—concentration of the parameter (bCOD, N, or P) in the synthetic wastewater feed at the start of each cycle and ***C*final**—concentration of the same parameter measured in the bioreactor effluent at the end of the 15-day HRT.

### 4.5. Statistical Analysis

All experiments were conducted in triplicate (*n* = 3). Prior to parametric analysis, data were tested for normality (Shapiro–Wilk test) and homogeneity of variance (Levene’s test), with all parameters meeting assumptions for parametric statistics (*p* > 0.05). Differences among the three bioreactor treatments (A, B, and C) for pH, chlorophyll *a*, TSS, VSS, VSS/TSS ratio, and nutrient removal efficiencies were evaluated using one-way ANOVA. When ANOVA indicated significant effects (*p* < 0.05), Tukey’s HSD post hoc test determined pairwise differences, with treatments sharing the same superscript letter denoting no significant difference at *p* ≤ 0.05. Pearson correlation analysis examined linear relationships among physicochemical parameters, with correlation strength interpreted as weak (|r| < 0.30), moderate (0.30 ≤ |r| < 0.70), or strong (|r| ≥ 0.70). All analyses were performed using OriginPro 2024 (Version 10.1), with significance levels denoted as ns (not significant, *p* > 0.05), * (*p* ≤ 0.05), ** (*p* ≤ 0.01), and *** (*p* ≤ 0.001).

## 5. Conclusions

This study demonstrates that phosphorus loading is a primary lever for engineering microalgal community structure in photobioreactors treating synthetic wastewater, driving predictable changes in composition—not merely biomass increases—and directly determining the system’s treatment performance. Increasing phosphorus concentration stimulated biomass production and induced a structural shift, with the same core taxa (*Chlorella* spp., *Scenedesmus* spp., and *Navicula* spp.) present across treatments but their relative abundances shifting significantly (bioreactor × genus interaction, *p* < 0.0001), culminating in the emergence and dominance of the cyanobacterium Anabaena spp. only under high-P conditions, along with algal aggregates and some zooplankton (e.g., ciliates such as Stylonichia). This phosphorus-driven community structuring was directly linked to a substantial enhancement in the system’s purification potential, with the highest phosphorus level yielding the greatest removal efficiencies for organic carbon (~85%), nitrogen (~78%), and phosphorus itself (~71%). A primary limitation of this work is its use of a synthetic, simplified wastewater and a laboratory-scale setup. Nevertheless, the findings provide a clear mechanistic basis for optimizing phosphorus management in practical applications. Specifically, the results indicate that controlled phosphorus dosing can be used as an engineering tool to steer community assembly and enhance nutrient removal in algal-based systems, particularly for decentralized treatment in arid regions. Future research should validate these phosphorus-driven effects in outdoor, pilot-scale systems treating real wastewater, where integrating these insights could lead to more robust, efficient, and resource-recovery-oriented treatment processes.

## Figures and Tables

**Table 1:** Algae and planktonic species observed in each bioreactor.

Bioreactors	Species/Type	Mean Abundance (Cells/L ± SD)	Zooplankton Presence
A	*Scenedesmus* spp.	3.0 ± 0.6 × 10^7^	*Stylonychia* spp., ciliates
	*Chlorella* spp.	6.2 ± 1.1 × 10^8^	
	*Navicula* spp.	5.0 ± 1.0 × 10^6^	
	Filamentous algae	2.5 ± 0.5 × 10^7^	
B	*Scenedesmus* spp.	2.2 ± 0.4 × 10^9^	*Stylonychia* spp., ciliates, with strong activity
	*Chlorella* spp.	4.5 ± 0.9 × 10^8^	
	*Navicula* spp.	3.0 ± 0.6 × 10^8^	
	Filamentous algae	5.0 ± 1.0 × 10^7^	
C	*Scenedesmus* spp.	2.0 ± 0.4 × 10^8^	*Stylonychia* spp., ciliates, with strong activity
	*Chlorella* spp.	7.0 ± 1.4 × 10^9^	
	*Navicula* spp.	1.2 ± 0.2 × 10^7^	
	Filamentous algae	6.0 ± 1.2 × 10^8^	

**Table 2:** Statistical analysis results of the algae and planktonic taxa abundance in each bioreactor.

	DF	Sum of Squares	Mean Square	F-Value	*p*-Value
A	2	2.00 × 10^19^	1.00 × 10^19^	3.96772	0.02959 *
B	3	3.89 × 10^19^	1.30 × 10^19^	5.14337	0.00547 **
Model	5	5.89 × 10^19^	1.18 × 10^19^	4.67311	0.00284 **
Error	30	7.56 × 10^19^	2.52 × 10^18^		
Corrected Total	35	1.35 × 10^20^			

*, significant at *p* ≤ 0.05; **, significant at *p* ≤ 0.01.

**Table 3:** The average data and statistical analysis of the physicochemical results at the outlet of the bioreactors.

Bioreactors	pH	Ch a (µg/L)	TSS (mg/L)	VSS (mg/L)	VSS/TSS (%)
A	7.47 ± 0.25 ^a^	11 ± 0.2 ^c^	12.26 ± 0.81 ^b^	8.57 ± 0.56 ^b^	69.93 ^a^
B	7.17 ± 0.16 ^a^	28.02 ± 4.53 ^b^	14.74 ± 0.63 ^b^	10.31 ± 0.45 ^b^	69.97 ^a^
C	7.73 ± 0.5 ^a^	43.9 ± 2.79 ^a^	22.8 ± 1.6 ^a^	15.96 ± 1.13 ^a^	70 ^a^
F	2.09	86	75.65	74.93	1.42
*p*	0.20 ^ns^	<0.0001 ***	<0.0001 ***	<0.0001 ***	0.313
CV%	4.5	11.12	6.61	6.66	0.03

^ns^: not significant; ***, significant at *p* ≤ 0.001. Means with the same letter are statistically equal (Tukey’s HSD at 0.05).

**Table 4:** Nutrient removal efficiencies in the three bioreactors.

Bioreactors	P Load (mg/L)	bCOD Removal (%)	N Removal (%)	P Removal (%)
A	1	68.5 ± 3.2 ^c^	62.1 ± 2.8 ^c^	45.3 ± 4.1 ^c^
B	10	76.8 ± 2.1 ^b^	70.5 ± 3.1 ^b^	65.7 ± 3.5 ^b^
C	20	84.9 ± 1.8 ^a^	77.8 ± 2.5 ^a^	71.2 ± 2.9 ^a^
F-value		98.45	85.72	92.13
*p*-value		<0.0001	<0.0001	<0.0001

Means with different superscript letters are significantly different (*p* < 0.05, Tukey’s HSD test). bCOD = biodegradable chemical oxygen demand (estimated from glucose input at 75% biodegradable fraction). Data are presented as mean ± standard deviation (*n* = 3 replicates).

## Data Availability

Data reported in the current study are contained within the article.

## Abbreviations

The following abbreviations are used in this manuscript:

C	Carbon (used in nutrient ratios such as C/N/P)
N	Nitrogen (used in nutrient ratios such as C/N/P)
P	Phosphorus (used in nutrient ratios such as C/N/P)
C/N/P	Carbon/nitrogen/phosphorus stoichiometric ratio
TSS	Total suspended solids
VSS	Volatile suspended solids
VSS/TSS	Ratio of volatile to total suspended solids
ANOVA	Analysis of variance
HSD	Honest significant difference
SD	Standard deviation (reported with mean values)
CV	Coefficient of variation
OD	Optical density (spectrophotometric absorbance)
bCOD	Biodegradable chemical oxygen demand
